# Effect of* Gelsemium elegans* and* Mussaenda pubescens*, the Components of a Detoxification Herbal Formula, on Disturbance of the Intestinal Absorptions of Indole Alkaloids in Caco-2 Cells

**DOI:** 10.1155/2017/6947948

**Published:** 2017-10-16

**Authors:** Yinghao Wang, Heshan Wang, Shuisheng Wu, Desen Li, Sainan Chen

**Affiliations:** College of Pharmacy, Fujian University of Traditional Chinese Medicine, Fuzhou 350108, China

## Abstract

*Gelsemium elegans* (GE) is a kind of well-known toxic plant. It can be detoxified by* Mussaenda pubescens* (MP), but the detoxification mechanism is still unclear. Thus, a detoxification herbal formula (GM) comprising GE and MP was derived. The Caco-2 cells monolayer model was used to evaluate GM effects on transporting six kinds of indole alkaloids of GE. The bidirectional transport studies demonstrated that absorbance percentage of indole alkaloids in GE increased linearly over time. But in GM, Papp (AP→BL) values of the most toxic members, gelsenicine, humantenidine, and gelsevirine, were lower than that of Papp (BL→AP) (*P* < 0.05). The prominent analgesic effect members, gelsemine and koumine, were approximately 1.00 in *γ* values. Nowhere was this increasing efflux more pronounced than in the case of indole alkaloids with N-O structure. In the presence of verapamil, the *γ* values of humantenidine, gelsenicine, gelsevirine, and humantenine were decreased by 43.69, 41.42, 36.00, and 8.90 percent, respectively. The *γ* values in presence of ciclosporin were homologous with a decrease of 42.32, 40.59, 34.00, and 15.07 percent. It suggested that the efflux transport was affected by transporters. Taken together, due to the efflux transporters participation, the increasing efflux of indole alkaloids from GM was found in Caco-2 cells.

## 1. Introduction

*Gelsemium elegans* Benth. (GE) belongs to the family Loganiaceae and is widely distributed in Northern America, East Asia, and Southeast Asia [1]. It is generally acknowledged that GE is a deadly poison. Many cases of poisoning have been reported for inadvertent ingestion of this plant [2]. However it has been used as a Chinese folk medicine for treatment of malignant tumors, pain, rheumatic arthritis, psoriasis, and immune function [2–4]. The phytochemical studies indicated that GE contains a series of active constituents such as alkaloids, triterpenes, iridoid, and phenylpropanoids [5–8]. Chief among them is the indole alkaloids, such as koumine, gelsevirine, humantenmine. In our previous works we had established the fingerprint of indole alkaloids component of dichloromethane extraction of GE and had found that indole alkaloids of GE had obvious analgesic effect in acetic acid stretching experiment of mice [9, 10]. Despite the benefits of the GE preparations to human health, it is not used abroad for its great toxicity. Although evaluation of GE toxic effects has been studied using animal models [2], there has not been any report on how to decrease its toxicity.

**Figure 1:**
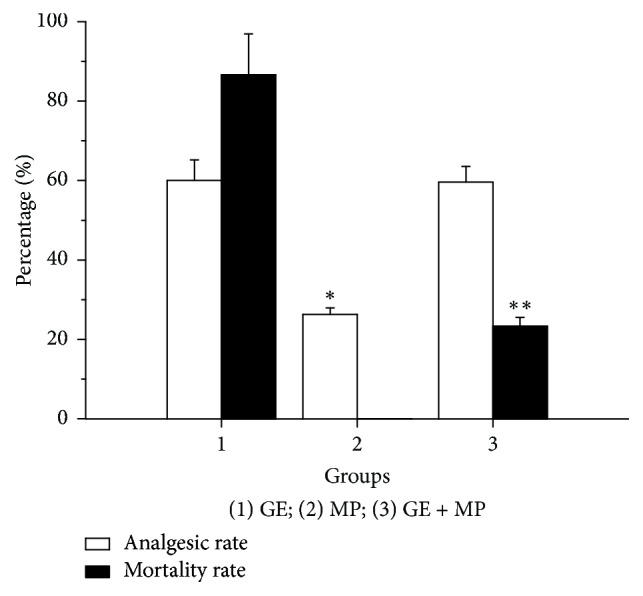
Toxicity/efficacy relation of GE with or without MP (*n* = 10). The administration dosage of GE dichloromethane extract in mice was 0.27 g·kg^−1^ and that of MP aqueous extract was 10.80 g·kg^−1^. The mortality rate was performed through the experiments of acute poisoning in mice. The analgesic rate was studied in mice by the experiments of acetic acid stretching. All results were expressed as $\overset{-}{X}\pm S$. ^*∗*^*P* < 0.05, ^*∗∗*^*P* < 0.01 when compared with GE group.

*Mussaenda pubescens* Ait. f. (MP) belongs to the Rubiaceae family and is widely distributed in valleys, shady hillsides, and shrub jungle of east, south, and southwest China. It has been used as a Chinese folk medicine for thousands of years. It is not only used to treat common cold, heatstroke, diarrhoea, and some inflammatory diseases, such as laryngopharyngitis and acute gastroenteritis [11], but also tried to detoxify mushroom poisoning, GE poisoning, and so on [12]. For instance, Fujianese recommend it as an antidote for GE poisoning. And what is really interesting is that GE tends to accompany growth of MP in nature. In our previous experiments we found that MP aqueous extract could lower the toxicity of GE dichloromethane extract [13]. Taking into account previous results obtained in the toxicity/efficacy studies with the two Chinese herbals, GE and MP (Figure 1), a new formula (GM) comprising GE dichloromethane extract and MP aqueous extract in the ratio of 1 : 40 was derived. But the detoxification mechanisms of GM in various aspects needed further studying.

Thus, the constituents of GE in the absence or presence of MP were comparatively analyzed by HPLC method. There were no significant differences in categories and contents of the constituents [9]. In light of the fact that there is no practical difference above of main ingredients between GE and GM, it led us to postulating that detoxification effect might be caused in the field of interfering with the constituent absorption. Therefore, the aim of this current study is to evaluate effect of the detoxification herbal formula on transport of indole alkaloids in Caco-2 cells monolayer. The specific objectives of the current studies include the following: (1) to study the bidirectional transport characteristics of indole alkaloids between GM extract and GE extract in Caco-2 cells monolayer; (2) to evaluate the effect of GM with or without verapamil (P-gp inhibitor) on transport of indole alkaloids; (3) to investigate the effect of GM in the absence or presence of ciclosporin (P-gp or MRP2 inhibitor) on transport of indole alkaloids.

## 2. Materials and Methods

### 2.1. Chemicals and Reagents

Ciclosporin and verapamil were purchased from Sigma Chemical Company (St. Louis, MO, USA). HPLC-grade water was obtained from a Milli-Q water purification system (Bedford, MA, USA). HPLC-grade acetonitrile and methanol were obtained from Merck (Merck, Darmstadt, Germany). HPLC-grade formic acid was obtained from Tedia (Fairfield, OH, USA). HPLC-grade ammonium acetate was obtained from Aladdin Co., Lid (Shanghai, China). Cell culture grade dimethyl sulfoxide (DMSO) was purchased from Sigma Chemical Company (St. Louis, MO, USA). Hank's balanced salt solution (HBSS), trypsin-EDTA, heat-inactivated fetal bovine serum (FBS), nonessential amino acid solution, and Dulbecco's modified Eagle's medium (DMEM) were purchased from Gibco Laboratories (Invitrogen Co, Grand Island, NY, USA). All other chemicals and solvents used were at least of analytical grade.

### 2.2. Preparation of Extracts

Fresh roots collection and dried powders extraction of GE were similar to those described previously [14]. The concentration of GE dichloromethane part was 0.05 g·ml^−1^. The entire plant collection and dried powders extraction of MP were similar to those described previously [13]. Then the extraction was extracted with petroleum ether, dichloromethane, and ethyl acetate, separately. Thereafter the residual aqueous part was collected. The concentration of MP aqueous part was 2.0 g·ml^−1^. Concentrations of GE dichloromethane part and MP aqueous part were all converted according to the material dosage. Two parts were filtered and evaporated under reduced pressure to obtain GE dichloromethane and MP aqueous extracts with typical yields of 0.67 ± 0.071% and 5.78 ± 0.20%, respectively. The compatibility proportion in GM was 1 : 40 (GE dichloromethane extract: MP aqueous extract).

### 2.3. Chemical Profiles of Extracts

**Figure 2:**
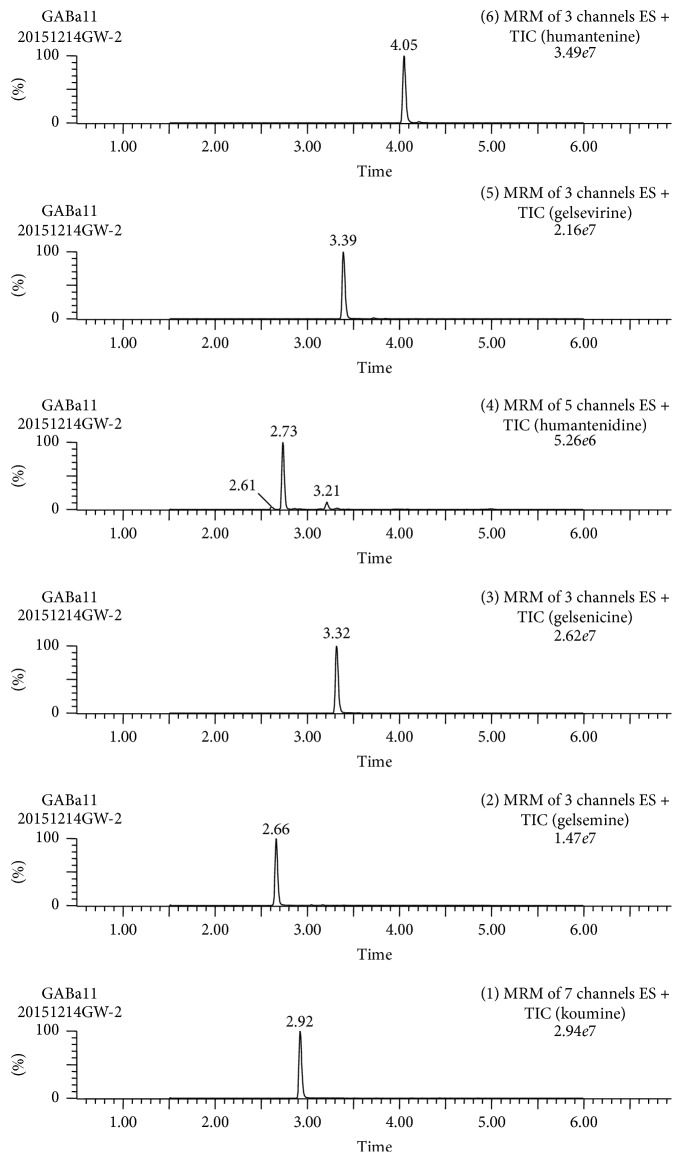
LC-MS/MS profile of determination of most of the ingredients in GM extract simultaneously.

**Figure 3:**
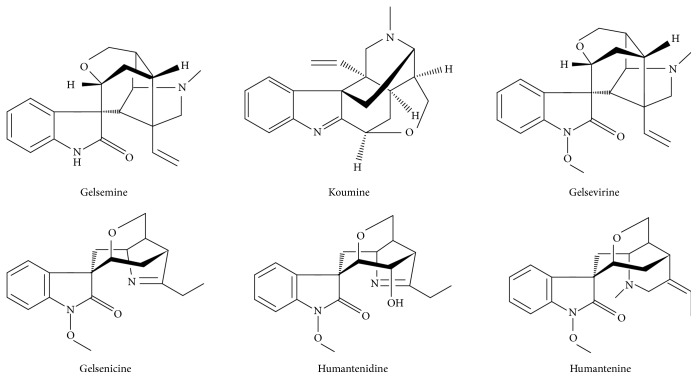
Structural formulae of analyte standards.

Identification of gelsemine, humantenidine, koumine, gelsenicine, gelsevirine, and humantenine in GE dichloromethane extract or GM extract was achieved by LC-ESI-MS/MS method. The analyses were performed using a triple-quadrupole Quattro micro mass spectrometer (Waters Corp., Milford, MA, USA) equipped with an electrospray ionization (ESI) interface to obtain structural information. The raw data were detected by Waters 2998 photodiode array detector and processed with Empower TM software. A Waters ACQUITY UPLC C18 column (100 × 2.1 mm, 1.7 *μ*m) (Waters, USA) was applied for all analyses. The mobile phase was composed of A (2 mM ammonium acetate-0.25% aqueous formic acid, v/v) and solvent B (acetonitrile) with a linear gradient elution: 0–0.5 min, 98-98% A; 0.5–1.5 min, 98–80% A; 1.5–3.0 min, 80–70% A; 3.0–4.0 min, 70–55% A; 4.0–4.1 min, 55–98%; 4.1–6.0 min, 98-98% A. The mobile phase flow rate was 0.3 ml·min^−1^, the column temperature was controlled at 40°C and sample injection volume was 2.0 *μ*l. ESI/MS/MS was operated using electrospray ionization in positive mode with the following parameters: capillary voltage, 3.5 kV; cone voltage, 50 V; desolvation temperature, 500°C; source temperature, 150°C; cone gas (nitrogen), 150 L·h^−1^; desolvation gas (nitrogen), 900 L·h^−1^. Under the mass spectrometric conditions, the maximum stable response of the precursor ions and the major product ions of the analytes were achieved. The maximal peak area in every product ion was chosen as quantitative ion pair. The rest were all qualitatively ion pairs. The chemical profiles of extracts were illustrated in Table 1 and Figure 2. The structures of six kinds of indole alkaloids were illustrated in Figure 3.

### 2.4. Cell Culture

Human colon adenocarcinoma cell lines (Caco-2) were obtained from the cell bank of Chinese Academy of Sciences (Beijing, China). Cells were routinely grown in DMEM supplemented with 10% (v/v) fetal bovine serum, 1% (v/v) glutamine, 1% (v/v) nonessential amino acid solution, and 100 U/mL penicillin-streptomycin solution under a humidified atmosphere of 5% CO_2_ at 37°C. The medium was replaced every 2 days after incubation. After reaching 80% confluence, cells were passaged at a split ratio of 1 : 5, using 0.05% trypsin-EDTA. For the transport experiments, the cells of passages were seeded at a density of 1.0 × 10^6^ cells/cm^2^ on permeable polycarbonate inserts in 6-well tissue culture plates (Transwell Polycarbonate Membrance Insert 3414, Corning, USA), which has a surface area of 4.2 cm^2^. Culture medium was changed every two days for 14 days and daily thereafter for the next 7 days. In our culture condition, cells monolayer, after seeding approximately between 19 and 21 days, usually was available for transport experiments.

The integrity of the cells monolayer was monitored by measuring transepithelial electrical resistance (TEER) across the cells monolayer. Briefly, the cells monolayer was washed three times with blank HBSS after culture medium was aspirated. Then TEER values of cells monolayer were measured at 37°C by using a Millicell-ERS meter (Millipore, USA). Cells monolayer was considered intact and suitable for transport assays when the TEER values were more than 550 Ω cm^2^ [15].

The TEER value was calculated with the following equation [16]:

(1) $$\begin{array}{c} \text{TEER}=\frac{\left({TEER}_{monolayer}-{TEER}_{blank}\right)}{A}, \end{array}$$

where TEER_monolayer_ is the resistance of the cells monolayer along with the filter membrane, TEER_blank_ is the resistance of the filter membrane, and* A* is the surface area of the membrane.

### 2.5. Cytotoxicity Assay

The cytotoxicity after giving medicine was determined through the reduction of MTT to formazan. The cells were seeded in 96-well plates (1.0 × 10^5^ cells/ml) and were incubated in the culture medium for 24 hours at 37°C under a humidified atmosphere of 5% CO_2_. Then the culture medium was removed and cells were bathed in culture medium supplemented with 0, 0.0156, 0.031, 0.062, 0.125, 0.25, or 0.50 mg·ml^−1^ extracts for 24 hours. Doses of the extracts, including GE and GM, were converted according to the GE material dosage. After incubation with 20 *μ*L of 5.0 g·L^−1^ MTT for 4 hours at 37°C, the MTT solution was aspirated. The formazan crystals were dissolved in 150 *μ*L DMSO by agitating the dishes on a micro oscillator. The absorbance (*A*) at 490 nm in each well was measured by using a microplate reader (MK3, Thermo, USA). The cytotoxicity was calculated according to (2). We further chose the concentration of cytotoxicity lower than 5% as security levels.

(2) $$\begin{array}{c} \text{\% cytotoxicity}=\left(\;1-\frac{A_{medication\;\;groups}}{A_{negative\;\;control\;\;groups}}\;\right)\times 100\%. \end{array}$$

### 2.6. Effects of the Detoxification Herbal Formula on Transport of Indole Alkaloids in Caco-2 Cells

#### 2.6.1. Bidirectional Transport Studies

The extracts, including GE and GM, were freshly prepared to the desired final concentration in DMSO. The final concentrations of DMSO in the incubation system were controlled within 0.1% (v/v) in all subsequent experiments. All transport experiments were conducted at 37°C unless specified otherwise. Transport of indole alkaloids across the Caco-2 cells monolayer was studied using monolayer of 19–21 days after seeding. Before the experiment, the monolayer was washed twice and preincubated with the blank HBSS for 1 h. Thereafter the HBSS solution on both sides of the cells monolayer was aspirated.

Transport studies were conducted in the absorptive direction (apical- (AP-) basolateral (BL)) and the efflux direction (BL-AP), separately. For determination of AP to BL transport, 2.5 mL of sample solutions was added to the AP side, respectively, and 2.5 mL of blank HBSS was added to the BL side. For determination of BL to AP transport, 2.5 mL of sample solutions was added to the BL side, respectively, and 2.5 mL of blank HBSS was added to the AP side. The cells monolayer was then put into a shaker at 50 rpm during the transport process. 400 *μ*L of samples was taken for LC-MS/MS analysis at 0, 1, 2, 3, and 4 h after incubation, followed by an immediate replacement of 400 *μ*L of the same solutions. At the end of the transport experiment, integrity of the monolayer was monitored by TEER value.

#### 2.6.2. Inhibition Transport Studies

As efflux transporters (P-gp and MRP2) are expressed on the apical side of Caco-2 cells, the absorption or efflux transport of indole alkaloids across this model was investigated in the presence of efflux transporters inhibitors. A selective P-gp inhibitor, verapamil (50 *μ*mol), was added to GM. 10 *μ*mol ciclosporin (a P-gp and MRP2 inhibitor) was also added to GM. The control group was set as well. After the preincubation, the different mixes were performed in Caco-2 cells monolayer. Transport studies were undertaken as mentioned above in the absorptive direction (AP-BL) and the efflux direction (BL-AP), separately. The samples were taken at 0, 1, 2, 3, and 4 h for LC-MS/MS analysis. The absorption and efflux transport apparent permeability (*P*_app_) of indole alkaloids were determined.

### 2.7. Determination of Analytes by LC-MS/MS

400 *μ*L of samples obtained from the Caco-2 cells transport assays was dried under nitrogen, and the residues were dissolved in 800 *μ*L of 50% methanol. Followed by centrifugation at 12,000 rpm for 15 min, the supernatants were directly analyzed with UPLC-ESI-MS/MS. Chromatography-mass spectrum conditions were set as described above. The method researches of LC-MS/MS were similar to those described previously [17]. Regression equations of gelsemine, humantenidine, koumine, gelsenicine, gelsevirine, and humantenine were *Y* = 23675*X* − 2864 (*r* = 0.9995), *Y* = 66331*X* + 13558 (*r* = 0.9995), *Y* = 6268*X* − 1503 (*r* = 0.9996), *Y* = 91160*X* − 10147 (*r* = 0.9998), *Y* = 32116*X* − 3951 (*r* = 0.9990), and *Y* = 36977*X* + 13830 (*r* = 0.9994), respectively.

### 2.8. Calculation and Data Analysis

All experiments were run in triplicate. Results were expressed as mean ± SD. The apparent permeability coefficients (*P*_app_) for both the absorptive direction (AP→BL) and the efflux direction (BL→AP) studies were calculated according to the following [18]:

(3) $$\begin{array}{c} P_{app}=\frac{\left(dQ/dt\right)}{\left(A\times C_{0}\right)}, \end{array}$$

where *dQ*/*dt* (*μ*g·min^−1^) is the drug permeation rate,* A* (cm^2^) is the filter surface area, and *C*_0_ (*μ*g·mL^−1^) is the initial concentration in the donor chamber at *t* = 0 min.

The efflux ratio (*γ*) was calculated with the following equation:

(4) $$\begin{array}{c} \gamma =\frac{P_{\text{app}\left(\text{BL-AP}\right)}}{P_{\text{app}\left(\text{AP-BL}\right)}}. \end{array}$$

The *γ* values are used to assess the effect of efflux transporters on the test compounds in the presence and absence of efflux transporters inhibitors.

## 3. Results

### 3.1. Evaluation of the Caco-2 Cells Monolayer Model

In our culture condition, compactness of Caco-2 cells monolayer was gradually rising. After seeding for approximately 20 days, TEER values were greater than 1000 Ω·cm^−2^ and tended to be constant. The results were shown in Table 2. It meant that Caco-2 cells had formed the complete and close cells monolayer and were available for transport experiments.

### 3.2. Cytotoxicity Assay of Extracts

**Figure 4:**
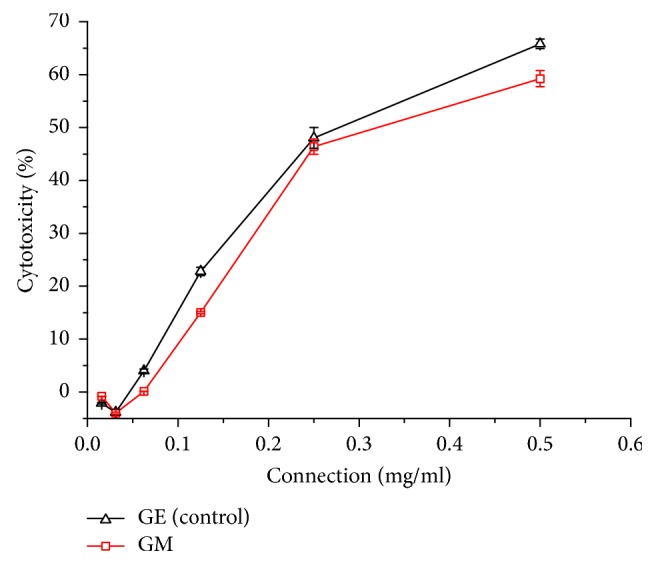
Effect on cytotoxicity in GE and GM samples ($\overset{-}{x}\pm s$, *n* = 3).

As shown in Figure 4, cytotoxicity of GM sample had trended down when compared with control sample. When concentration was, respectively, 0.062 mg·ml^−1^ at GE and 0.062 mg·ml^−1^ at GM (namely, 8 times diluted concentration as compared with 0.50 mg·ml^−1^), cytotoxicity was all below 5%. In other words, cell survival rates were over 95% in this concentration. Considering an extremely low rate of cell injury and need of detectable concentration (see reference [17]), we chose 8 times diluted concentration as the relative security concentration.

### 3.3. Bidirectional Transport Studies

**Figure 5:**
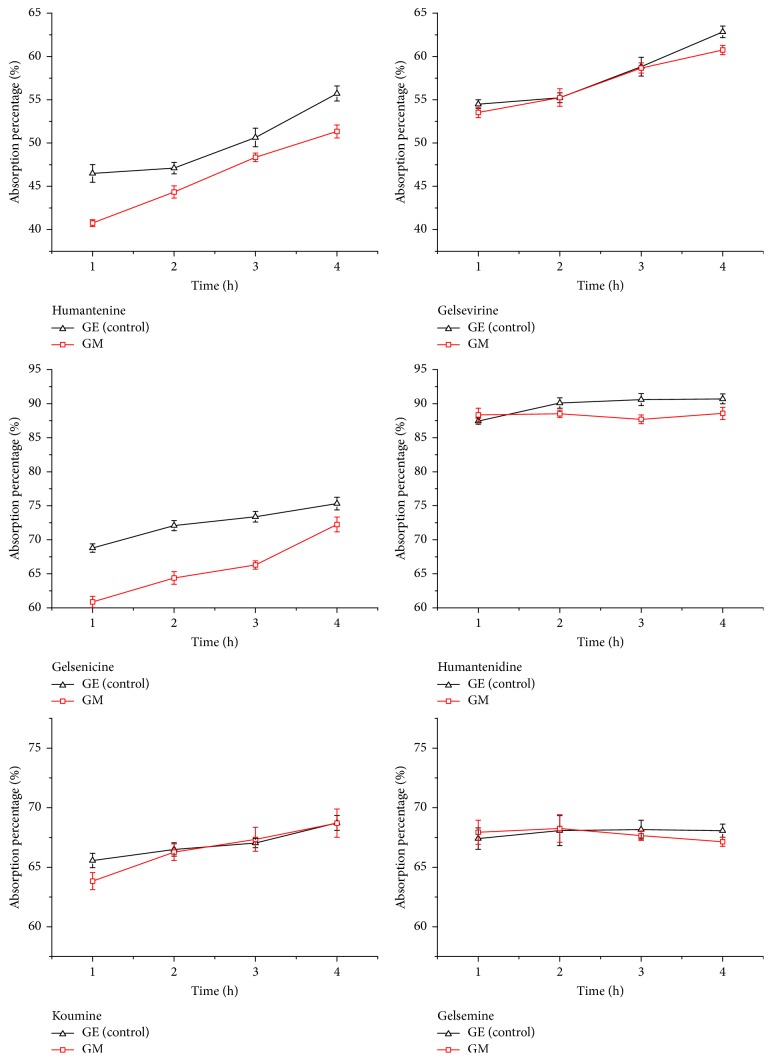
The absorbance percentage of six indole alkaloids in GE and GM samples during 4 h from apical to basolateral direction in the Caco-2 cells monolayer($\overset{-}{x}\pm s$, *n* = 3).

Due to detoxification of compatibility of GE and MP in traditional Chinese medicine, the absorptive transport of indole alkaloids was examined.  Figure 5 shows the results of absorbance percentage of six indole alkaloids from GE and GM samples inner 4 h in Caco-2 cells monolayer. It was observed that absorbance percentages of indole alkaloids of GE sample were all more than 40% in 1 h and increased linearly over time. Humantenidine had the highest absorbance percentage among all indole alkaloids. It suggested that indole alkaloids from GE were well absorbed compounds. But after combination treatment of GE and MP, absorbance percentages of humantenine and gelsenicine were in a big decrease. Their decrements were about 12%. Absorbance percentages of gelsevirine had lost over 3%. As can be seen above, the herbal formula might mean less indole alkaloids absorbance in Caco-2 cells monolayer. But whether the detoxification had anything to do with efflux transport needs to be studied.

**Figure 6:**
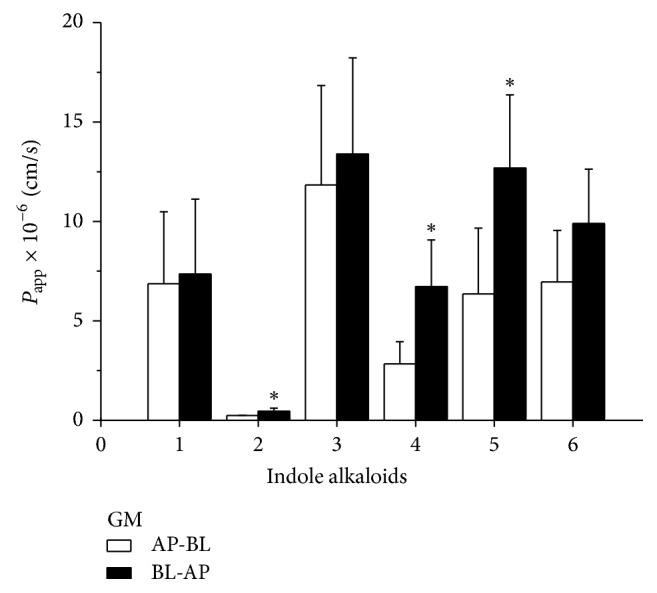
The apparent permeability coefficients of bidirectional transport of six indole alkaloids in GM (*n* = 3). All results were expressed as $\overset{-}{X}\pm S$. ^*∗*^*P* < 0.05 when compared with absorptive direction. (1) Gelsemine; (2) humantenidine; (3) koumine; (4) gelsenicine; (5) gelsevirine; (6) humantenine.

As indicated in Figure 6, *P*_app_ (AP→BL) values of six indole alkaloids were lower than that of *P*_app_ (BL→AP). There was significant difference in the values of humantenine, gelsenicine, and gelsevirine (*P* < 0.05). It implied that the herbal formula could increase the efflux of indole alkaloids in the small intestine. But the cause of increasing efflux of indole alkaloids was still unclear. Therefore the detoxification herbal formula effects on efflux transporters were studied in the Caco-2 cells monolayer.

### 3.4. Inhibition Transport Studies

#### 3.4.1. Effect of GM with or without Verapamil on the Transport of Six Indole Alkaloids

To determine the effect of the detoxification herbal formula, the transport assays of GM + verapamil and GM (control group) were carried out. The *P*_app_ coefficients of indole alkaloids across Caco-2 cells monolayer in the AP→BL and BL→AP directions were presented in Table 3. Then the efflux ratio (*γ*) was calculated in Table 4. When verapamil was used in combination with GM, the *P*_app_ values of 6 kinds of indole alkaloids were all increased whether in AP-BL direction or in BL-AP direction. (*P* < 0.01, Table 3). However, the efflux ratio was on the decline as compared with GM sample (Table 4). The efflux rate of humantenidine was decreased by 43.69 percent, and that of gelsenicine, gelsevirine, and humantenine was decreased by 41.42, 36.00, and 8.90 percent, respectively. These results suggested that the efflux transport of indole alkaloids was affected by P-gp inhibitor; in the other words, P-gp probably participated in the efflux transport of indole alkaloids in the detoxification herbal formula.

#### 3.4.2. Effect of GM with or without Ciclosporin on the Transport of Six Indole Alkaloids

To determine the effect of the detoxification herbal formula, the transport assays of GM + ciclosporin and GM (control group) were carried out. The *P*_app_ coefficients of indole alkaloids across Caco-2 cells monolayer in the AP→BL and BL→AP directions were presented in Table 5. Then the efflux ratio (*γ*) was calculated in Table 6. When ciclosporin was used in combination with GM, the *P*_app_ value of 6 kinds of indole alkaloids was all increased whether in AP-BL direction or in BL-AP direction (*P* < 0.01, Table 5). However, the efflux ratio was on the decline as compared with GM sample (Table 6). The efflux rate of humantenidine was decreased by 42.32 percent, and that of gelsenicine, gelsevirine, and humantenine was decreased by 40.59, 34.00, and 15.07 percent, respectively. These results suggested that the efflux transport of indole alkaloids was affected by ciclosporin; in other words, MRP2 and P-gp probably participated in the efflux transport of indole alkaloids in the detoxification herbal formula.

## 4. Discussion

**Figure 7:**
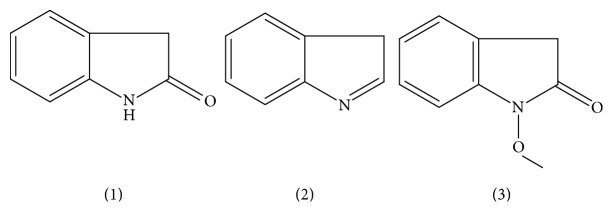
Mother nucleus structure of six indole alkaloids. 1: mother nucleus of gelsemine; 2: mother nucleus of koumine; 3: mother nucleus of humantenidine, gelsenicine, gelsevirine, or humantenine.

Although chemical composition of GE has been extensively studied for its prominent medical activities since 1980s [5, 7, 19, 20], there is lack of comprehending information regarding its synergism and attenuation. The present research provides the first investigation of the intestinal absorption and efflux characteristics of six kinds of indole alkaloids using Caco-2 cells monolayer model. The aim is to evaluate effects of the detoxification herbal formula on transport of indole alkaloids. Our results found that absorbance percentage of indole alkaloids from GE sample increased linearly over time (Figure 5), showing that active transport is the dominant transport process for indole alkaloids in Caco-2 cells monolayer. But after GE combined with MP, namely the detoxification herbal formula (GM), the efflux *P*_app_ of humantenidine, gelsenicine, and gelsevirine was approximately 2.0 times higher than that of the absorptive *P*_app_ (Figure 6), suggesting that efflux transport was preponderant. It had been reported that the most toxic member of indole alkaloids is gelsenicine, and then humantenidine [21]. As mentioned, the *γ* values of humantenidine, gelsenicine, and gelsevirine in GM sample were over 2.00. Thus, the decrease in bioavailability of these indole alkaloids was of such a magnitude to cause a reduction in the toxicity of GM. Nowhere was this detoxification mechanism more pronounced than in the case of indole alkaloids with N-O structure (Figure 7). It might be because of the increasing polarity caused by N-O structure. On the other hand, the prominent analgesic effect members of indole alkaloids, gelsemine and koumine [21], were approximately 1.00 in *γ* values, suggesting an equivalence relation between absorption and efflux. The result was a good explanation of toxicity/efficacy relation of GE with or without MP as shown in Figure 1.

To determine the effect of the detoxification herbal formula, the transport assays of GM with or without P-gp inhibitor, verapamil, were performed. As compared with GM sample, the efflux rates of humantenidine, gelsenicine, gelsevirine, and humantenine were all decreased (Tables 3 and 4). These results suggested that the efflux transport of indole alkaloids was affected by verapamil. Nevertheless, this result cannot lead to the conclusion that the P-gp was involved in the uptake of indole alkaloids because the substrates of efflux transporters cross each other.

**Figure 8:**
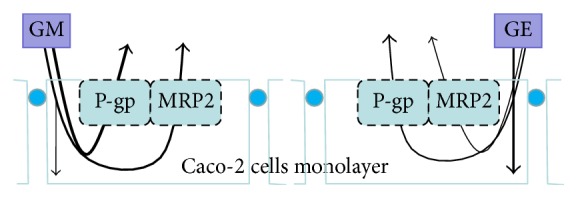
The transport model of GE and GM mediated by transporters in the Caco-2 cells.

Therefore, ciclosporin (both P-gp inhibitor and MRP2 inhibitors) was carried out in the process of transport assays to confirm the effect of efflux transporters. It can be seen from Tables 5 and 6 that the efflux rates of humantenidine, gelsenicine, gelsevirine, and humantenine were all decreased as compared with GM samples. These results suggested that the efflux transport of indole alkaloids from GM sample was affected by ciclosporin. Thus, our results suggested that P-gp participated in the efflux transport of indole alkaloids and MRP2 might be involved in the efflux transport of indole alkaloids. The conclusions of the present study are summarized in Figure 8. Taken together, due to the efflux transporters participation, the reducing absorption and increasing efflux of indole alkaloids from the detoxification herbal formula were found in Caco-2 cells monolayer model.

## Figures and Tables

**Table 1:** Precursor/product ion pairs and parameters for MRM of compounds used in this study.

Analyte	GE dichloromethane extract	GM extract	Retention time (min)	[M+H]^+^*(m/z)*	MRM transition (precursor→product)	Peak area of product	Cone voltage (V)	Collision energy (eV)
Gelsemine	+	+	2.65	323.17	323.2→70.0^*α*^	97565	30	30
					323.2→195.0	17501	30	30
					323.2→236.0	40783	30	25
Humantenidine	+	+	2.72	343.20	343.2→312.1^*α*^	10351	30	16
					343.2→281.3	1627	30	23
					343.2→108.1	1971	30	25
Koumine	+	+	2.91	307.17	307.2→180.0^*α*^	78147	20	40
					307.2→167.0	52038	20	50
					307.2→204.0	56263	20	50
Gelsenicine	+	+	3.30	327.17	327.2→296.0^*α*^	193147	30	25
					327.2→265.0	41147	30	18
					327.2→108.0	23742	30	30
Gelsevirine	+	+	3.37	353.10	353.1→322.0^*α*^	264565	20	18
					353.1→110.3	29739	20	30
					353.1→259.9	22903	20	30
Humantenine	+	+	4.02	355.11	355.1→309.1^*α*^	426987	20	35
					355.1→178.3	72919	20	25
					355.1→122.1	142393	20	25

^*α*^Quantitative ion pair.

**Table 2:** TEER value of Caco-2 cells monolayer ($\overset{-}{x}\pm s$, *n* = 3).

	6-well plates					
	1	2	3	4	5	6
*R* (Ω)	240.0 ± 21.1	246.7 ± 0.6	251.7 ± 18.1	255.3 ± 7.2	249.7 ± 17.0	255.3 ± 16.2
*A* (cm^−2^)	4.2	4.2	4.2	4.2	4.2	4.2
TEER (Ω·cm^−2^)	1008.0 ± 48.4	1036.0 ± 2.4	1057.0 ± 76.2	1072.4 ± 30.4	1048.6 ± 71.6	1072.4 ± 67.9

**Table 3:** Effect of GM with or without verapamil on *P*_app_ value of six indole alkaloids.

Indole alkaloids	GM+ verapamil		GM (control group)	
	*P*_app (AP-BL)_ (cm/s × 10^−6^)	*P*_app (BL-AP)_ (cm/s × 10^−6^)	*P*_app (AP-BL)_ (cm/s × 10^−6^)	*P*_app (BL-AP)_ (cm/s × 10^−6^)
Gelsemine	30.29 ± 7.45^a^	33.16 ± 8.00^b^	6.83 ± 2.64	7.33 ± 2.77
Humantenidine	3.14 ± 1.19^a^	6.17 ± 0.57^b^	0.14 ± 0.044	0.41 ± 0.05
Koumine	347.81 ± 67.39^a^	415.80 ± 80.47^b^	11.82 ± 4.01	13.38 ± 3.84
Gelsenicine	86.60 ± 15.75^a^	121.67 ± 26.05^b^	2.80 ± 0.11	6.69 ± 1.35
Gelsevirine	392.08 ± 58.13^a^	503.33 ± 93.03^b^	6.33 ± 2.31	12.68 ± 2.67
Humantenine	436.58 ± 71.15^a^	580.33 ± 108.86^b^	6.93 ± 1.59	9.87 ± 1.75

AP-BL is *P*_app_ in the apical-to-basolateral direction and BL-AP is *P*_app_ in the basolateral-to-apical direction. Data are expressed as mean ± SEM (*n* = 3). ^a^Significantly different from control of *P*_app (AP-BL)_ (*P* < 0.01). ^b^Significantly different from control of *P*_app (BL-AP)_ (*P* < 0.01).

**Table 4:** The *γ* values of GM across Caco-2 cells monolayer in the absence or presence of verapamil.

Samples	Gelsemine	Humantenidine	Koumine	Gelsenicine	Gelsevirine	Humantenine
*γ*_GM_	1.07	2.93	1.13	2.39	2.00	1.46
*γ*_GM+ verapamil_	1.09	1.65	1.19	1.40	1.28	1.33

**Table 5:** Effect of GM with or without ciclosporin on *P*_app_ value of six indole alkaloids.

Indole alkaloids	GM+ ciclosporin		GM (control group)	
	*P*_app (AP-BL)_ (cm/s × 10^−6^)	*P*_app (BL-AP)_ (cm/s × 10^−6^)	*P*_app (AP-BL)_ (cm/s × 10^−6^)	*P*_app (BL-AP)_ (cm/s × 10^−6^)
Gelsemine	35.56 ± 7.63^a^	39.11 ± 8.91^b^	6.83 ± 2.64	7.33 ± 2.77
Humantenidine	4.48 ± 1.06^a^	7.57 ± 0.36^b^	0.14 ± 0.044	0.41 ± 0.05
Koumine	440.14 ± 93.35^a^	523.50 ± 91.53^b^	11.82 ± 4.01	13.38 ± 3.84
Gelsenicine	101.06 ± 28.35^a^	143.30 ± 10.78^b^	2.80 ± 0.11	6.69 ± 1.35
Gelsevirine	464.20 ± 93.80^a^	613.70 ± 56.77^b^	6.33 ± 2.31	12.68 ± 2.67
Humantenine	515.48 ± 101.00^a^	637.00 ± 90.12^b^	6.93 ± 1.59	9.87 ± 1.75

AP-BL is *P*_app_ in the apical-to-basolateral direction and BL-AP is *P*_app_ in the basolateral-to-apical direction. Data are expressed as mean ± SEM (*n* = 3). ^a^Significantly different from control of *P*_app (AP-BL)_ (*P* < 0.01). ^b^Significantly different from control of *P*_app (BL-AP)_ (*P* < 0.01).

**Table 6:** The *γ* values of GM across Caco-2 cells monolayer in the absence or presence of ciclosporin.

Samples	Gelsemine	Humantenidine	Koumine	Gelsenicine	Gelsevirine	Humantenine
*γ*_GM_	1.07	2.93	1.13	2.39	2.00	1.46
*γ*_GM+ ciclosporin_	1.10	1.69	1.19	1.42	1.32	1.24
